# Associations between postural orientation errors in patients undergoing rehabilitation for ACL reconstruction and future patient-reported outcomes: An explorative study

**DOI:** 10.1016/j.jsampl.2023.100039

**Published:** 2023-09-24

**Authors:** Anna Cronström, Eva Ageberg, Erika Zeraidi, Julia Larsson, Jenny Nae

**Affiliations:** a Department of Health Sciences, Lund University, Box 117, 221 00 Lund, Sweden; b Department of Community Medicine and Rehabilitation, Umeå University, Umeå, Sweden

**Keywords:** Anterior cruciate ligament, Knee injuries, Postural orientation, Patient reported outcome measures

## Abstract

**Objective:**

To investigate associations between postural orientation errors (POEs) in patients undergoing rehabilitation for anterior cruciate ligament reconstruction (ACLR) and patient-reported outcomes (PROMs) at 2-year follow-up.

**Design:**

Prospective cohort study.

**Methods:**

Fifty-three participants (mean (SD) 27 (6.5) years, 24 women), (mean (range) 7 (4–10) months post ACLR) were included. At baseline, all participants were visually assessed for POEs using a validated test battery. The POE subscales Activities of Daily Living and Sport were used in the analysis. At 2-years, the following PROMs were collected: Global knee function, Knee injury and Osteoarthritis Outcome Score, ACL Quality of Life (QoL), Knee Self-Efficacy Scale (K-SES), and ACL Return-to-Sport after Injury scale.

**Results:**

Twenty-one participants answered the questionnaires at 2 years (7 women and 14 men). Worse baseline POE Sport was associated with worse scores on K-SES (r_s_ ​= ​–0.435, p ​≤ ​0.049) and ACL-QoL (r_s_ ​= ​−0.467 to −0.576, p ​≤ ​0.038) at follow-up. No statistically significant associations were observed between POEs and the other PROMs.

**Conclusion:**

Postural orientation during the rehabilitation phase may be important for future knee self-efficacy and knee-related QoL after ACLR. Given the small population and low response rate, this result needs to be confirmed in future research.

## Practical implications

This is the first longitudinal study, investigating possible associations between clinical assessments of postural orientation errors (POEs) during the rehabilitation phase after ACL reconstruction and future patient-reported outcome measures.

Although this was an explorative study, the significant associations between worse POEs at baseline and worse K-SES and ACL-QoL 2 years later indicate some importance of postural orientation for future knee self-efficacy and worse knee-related quality of life.

Given the small population, this result needs to be confirmed in future research.

## 1 Introduction

Injury to the anterior cruciate ligament (ACL) is devastating and associated with significant consequences, such as persisting functional limitations, decreased physical activity, impaired long-term quality of life (QoL) and a considerable risk of sustaining a second knee injury [1,2]. Rehabilitation may include non-surgical interventions as well as surgical ACL reconstruction (ACLR) with return to sport (RTS) as the main goal [3]. Clinical rehabilitation guidelines typically recommend a goal-based approach including an extensive test battery of muscle strength, hop performance, movement quality, and patient-reported outcomes to assess progression of the rehabilitation program and appropriated time point of RTS after ACL injury [1,4]. Movement quality, specifically, is suggested to add important information regarding neuromuscular function that may not be captured by commonly used RTS criteria, such as muscle strength and hop performance [1,4].

Movement quality, as assessed by postural orientation (the ability to stabilize the body segments in relation to each other and to the environment during weight-bearing movements) [5] can be evaluated using two-dimensional or three dimensional (3D) movement analysis systems or by using a more clinically applicable method, such as visual assessment. Individuals with ACL injury appear to have worse postural orientation compared with both healthy non-injured individuals and to the contra-lateral leg, [6,7] deficiencies that may persist decades after the injury [8,9]. More importantly, worse postural orientation seems to be related to an increased risk of second ACL injury to either knee after RTS [10,11], which further highlights the importance of incorporating postural orientation as a standard RTS criteria in this population.

Another goal of the rehabilitation, in addition to RTS, is to sustain long-term patient-reported function and QoL [1]. While there is evidence that factors such as physical activity level, muscle function, fear of re-injury and sustaining additional knee injuries may contribute to worse patient-reported outcomes in these patients [1,12], conflicting results have been reported for the relationship between movement quality and patient-reported outcomes [13,14]. In a cross-sectional study, no associations were found between a test battery for visually assessed postural orientation errors (POEs), including both daily activities and more demanding tasks such as hop tasks, and several patient-reported outcome measures (PROMs) at 7 months post ACLR [13]. On the other hand, Flosadottir et al. [12] found an association between worse score on the test battery for assessment of “*substitution patterns”* [6] during functional movements (e.g., body-weight-altering and knee flexion), and worse Knee injury and Osteoarthritis Outcome score (KOOS) (subscales pain, sport/recreation, QoL) at 3 years post ACL injury. However, the test for *substitution patterns* includes other aspects in addition to postural orientation, such as balance, body weight distribution and stride length, thus comparison between the two studies is difficult.

To our knowledge, only one study has investigated longitudinal associations between postural orientation and patient-reported outcomes in patients with ACL injury [15]. Ithurburn et al., reported an association between 3D assessed trunk and knee flexion asymmetry during a single-leg drop landing at the time of RTS and worse KOOS scores (pain and QoL) two years later [15]. Additionally, there are no studies that have investigated the association between visual assessment of postural orientation in patients with ACL injury during the rehabilitation phase and future patient-reported outcomes. Such knowledge would be important to guide rehabilitation protocols and to improve long-term patient-reported outcomes in this group of patients. Therefore, the aim of this prospective study was to investigate possible associations between POEs in the rehabilitation phase and PROMs at 2-year follow-up in individuals with ACLR.

## 2 Methods

This is a secondary explorative prospective analysis of a cohort study, with the original purpose to investigate measurement properties of a newly developed test battery for assessing POEs [16]. This study adheres to the STROBE statement (www.strobe-statement.org).

The cohort included in this study is thoroughly described in a previous publication [16]. Fifty-three participants (mean (SD) 27 (6.5) years, 24 women) were assessed for POEs at baseline (mean (SD) 27 (7) weeks after ACLR). Inclusion criteria were: age 18–39 years, ≥4 months and ≤10 months post ACLR, and started with rehabilitation exercises including jumping and change of direction. Exclusion criteria were: completed rehabilitation, medial collateral ligament injury grade 3, or other physical impairments or diseases that affected knee function. The study was approved by the Regional Ethical Review Board in Lund, Sweden (2018/14) and all participants gave their written informed consent.

All patient characteristics and data for POEs were collected at baseline. Pre-injury and baseline activity level were assessed with the Tegner Activity Scale (1–10, low to high knee demanding activity) [17]. At 2-year follow-up, an invitation to answer questionnaires regarding knee function, QoL, and readiness to RTS were sent out using an electronic data capture tool (REDCap, hosted by Lund University) [18] to all participants.

POEs at baseline were assessed using a valid and reliable test battery consisting of 5 standardized functional tasks performed on their injured leg as described in Table 1; single-leg mini squat, stair descending, forward lunge, single-leg hop for distance and side-hop [16,19]. Each task was video recorded from a frontal view (Oqus color video camera (2c-series), 30 ​Hz, version 2.12, Gothenburg, Sweden).

**Table 1:** Description of the functional tasks in the test battery for visual assessment of Postural Orientation Errors (POEs).

Task	Description	Repetitions	POEs assessed
*Single-leg mini squat*	A bench was adjusted so that the depth of the squat was approximately 60°. The patient was standing in front of the bench on 1 leg, with arms alongside the body. The patient was instructed to flex the knee until they lightly touched the bench with their buttocks and return to extension.	5 repetitions	During the entire movement.
*Stair descending*	The patient was standing on a step board with arms alongside the body. The patient was instructed to step down with the non-injured leg and take a few steps on the floor.	5 repetitions	From starting position until the foot left the step board, on the loading leg.
*Forward lunge*	The patient was standing with feet hip-width apart and took a long stride forward with the ACLR leg, flexed the knee to approximately 90°, and pushed back with the front leg to the starting position.	3 repetitions	From the first contact with the floor until maximum knee flexion on the front leg.
*Single-leg hop for distance*	The patient was standing on one leg with the other leg lifted from the floor. Instructions were to jump forward as far as possible, taking off and landing on the same foot. The use of arms was allowed. The task was approved if the balance was maintained for 3 ​s after landing. 3 approved landings were recorded.	3 approved landings	From first contact with the floor to 3–4 ​s after landing.
*Side-hop*	The patient was standing on one leg beside 2 parallel lines, 30 ​cm apart. Instructions were to jump from side to side on the test leg over the lines at a self-selected pace, starting with a lateral jump. The use of arms was allowed.	7 landings	From when the patella reached its lowest point during 3 medial and 3 lateral landings, the last landing was not assessed.

POEs ​= ​postural orientation errors, ACLR ​= ​anterior cruciate ligament reconstruction.

One physical therapist (JN) performed all evaluations of POEs on the video recordings. Six segment-specific POEs were specifically graded: foot pronation, knee medial-to-foot position, femur medial to shank, femoral valgus, deviation of pelvis in any plane, and deviation of trunk in any plane. Each segment-specific POE was rated from 0 (good) to 2 (poor), in line with a valid and reliable scoring system (inter-rater, weighted kappa ​= ​0.429–0.875) [16,19]. A score of 3 was given if the participant could not perform the exercise in any way similar to the expected performance. The POEs were divided into two subscales: the POE subscale Activity of Daily Living (ADL), including single-leg squat, Stair descending, and forward lunge and the POE subscale Sport, including single-leg hop for distance and side-hop. Each subscale was calculated to a minimum score of 0 (good) and a maximum score of 100 (poor). For a more detailed description of the scoring-system, and which segment-specific POEs that were rated for each of the 6 tasks (see Appendix A, Table 1).

At the 2-year follow-up, data for the following PROMs were collected: Knee Self-Efficacy Scale (K-SES), the KOOS, the Anterior Cruciate Ligament-Quality of Life (ACL-QoL), Global Knee Function, and Anterior Cruciate Ligament–Return to Sport after Injury (ACL-RSI). In addition, patients were asked to report any new ACL-injuries.

K-SES is a validated and reliable self-efficacy questionnaire that consists of two subscales, Present and Future. The subscale Present was used in the current analysis. The questionnaire is ACL injury specific and each item is scored on a 11 grade Likert scale from 0 to 10, where 10 indicates strong self-efficacy and 0 indicates poor self-efficacy [20].

KOOS is a valid and reliable PROM specifically designed for assessing knee specific symptoms in individuals with knee injuries and knee osteoarthritis including 5 subscales: Pain, Symptom, ADL, Sport/Recreation and QoL. Scores are given on a scale graded 0–4, which are thereafter converted into a 0–100 scale for each category, where 100 indicates no problems and 0 indicates maximum problems [21].

ACL-QoL was developed to evaluate QoL after ACL injury. The questionnaire is divided into 5 domains: symptoms and physical complaints, work-related concerns, recreation and sport concerns, lifestyle concerns, and social and emotional concerns. A total of 32 items are included and graded on a visual analogue scale (VAS) 0–100, where 100 is representing no problems and 0 is representing maximum problems. The results are summarized on a 100-grade scale where each question weighs equally. In this study, the domains recreation and sport, lifestyle, and total score were used in the analysis.

Global Knee Function was evaluated using the Numeric Rating Scale (1–100 were 1 ​= ​best and 100 ​= ​worst) [22] to assess the patients’ perceived overall knee function.

The ACL-RSI quantifies psychological factors associated with psychological readiness to RTS after injury. The scale covers emotions, confidence in performance, and risk appraisal and is measured on a 0–100 scale (0 indicates low readiness and 100 indicate high readiness) [23].

All data were analyzed using SPSS version 25 (IBM Corporation, New York, USA). Comparisons of characteristics and POEs at baseline between those who responded to the follow-up questionnaires and those who were lost to follow-up were performed using the independent t-test (continuous data), Mann–Whitney U test (ordinal data) or chi-square test (nominal data). The Spearman's rank correlation coefficient was used to determine any associations between POEs at baseline and PROMs at the 2-year follow-up. The following thresholds for the correlation coefficients were used: ≥0.1 ​= ​small, ≥0.3 ​= ​moderate, ≥0.5 ​= ​large and ≥0.7 ​= ​very large correlation [24]. Statistical significance was determined by p-values ≤0.05. Since this was an exploratory study, no adjustments for multiple comparisons were made [25].

## 3 Results

Out of the 53 participants invited to participate in the follow-up, 21 (40%) answered the questionnaires at 2 years (7 women and 14 men). No statistically significant differences in baseline characteristics or baseline POEs were observed between those who responded at follow-up and those who did not (see baseline characteristics and baseline POEs in Table 2). None of the participants reported any new ACL-injuries at follow-up. Descriptive data for the different patient-reported outcome variables at follow-up are presented in Table 3.

**Table 2:** Baseline data for those who responded and those who were lost to follow-up.

	Responded (n ​= ​21)	Lost to follow-up (n ​= ​32)	p-value
Age^a^	26 (6.9)	27 (6.3)	0.67
BMI^a^	25 (3)	24.5 (3.4)	0.68
Time since surgery^a^ (weeks)	28.6 (6.7)	27 (6.4)	0.44
Women, n (%)^b^	7 (33)	17 (53)	0.16
Tegner before injury^c^	8 (7–9)	8 (5–9)	0.70
Tegner at baseline^c^	4 (2.5–5)	3 (2–4)	0.34
POE subscale ADL^c^	17 (11–23.5)	22 (11–31)	0.29
POE subscale Sport^c^	31 (12.5–34.5)	31 (19–36)	0.69

BMI = Body Mass Index, POE ​= ​postural orientation error, ADL ​= ​Activities of Daily Living.

a Mean (SD), Independent sample t-test.

b Chi-square test.

c Median (quartiles), Mann Whitney U-test.

**Table 3:** Descriptive statistics of Patient Reported Outcome Measures (PROM) at follow-up.

PROM	Mean (SD) at follow-up (n ​= ​21)
K-SES	7.6 (2.1)
KOOS - ADL	95.3 (6.1)
KOOS - Sport	68.6 (7.6)
KOOS - QoL	62.1 (24.7)
ACL - QoL - Lifestyle	62.3 (29.3)
ACL - QoL - Sport	52.3 (31.1)
ACL - QoL Total score	59.3 (23.1)
Global Knee Function	29.1 (24.8)
ACL - RSI	45.6 (30.4)

K-SES = Knee Self-Efficacy Scale, KOOS = Knee injury Osteoarthritis Outcome Score, ADL ​= ​Activity of Daily Living, QoL ​= ​quality of life, ACL-RSI ​= ​Anterior Cruciate Ligament–Return to Sport after Injury.

Moderate to large statistically significant associations were found between higher baseline scores on the POE subscale Sport and worse scores on K-SES (r_s_ ​= ​−0.435, p ​= ​0.049) and all ACL QoL subscales (r_s_ ​= ​−0.467 to −0.576, p ​≤ ​0.038) at follow-up. No significant associations were observed between POE scores and Global Knee Function, the KOOS subscales, or ACL-RSI, or between POE subscale ADL and any of the PROMs (p ​≥ ​0.081) (Table 4). Scatterplots for all correlations are provided in Appendix B (Figs. 1–22).

**Table 4:** Spearman's Rank Correlation Coefficient (r_s_) between baseline POE scores and PROMs at 2-year follow-up (n ​= ​21).

PROM	POE subscale Sport			POE subscale ADL		
	r_s_	95% CI	p-value	r_s_	95% CI	p-value
K-SES	**−0.435**	−0.736; −0.009	**0.049**	−0.369	−0.698; 0.088	0.100
KOOS - pain	−0.381	−0.705; 0.074	0.088	−0.152	−0.557; 0.313	0.511
KOOS - symptoms	−0.389	−0.710; 0.065	0.081	−0.343	−0.682; 0.118	0.088
KOOS - ADL	−0.215	−0.601; 0.252	0.349	−0.360	−0.692; 0.099	0.109
KOOS – sport/recreation	−0.274	−0.639; 0.192	0.230	−0.247	−0.622; 0.220	0.280
KOOS - QoL	−0.360	−0,692; 0.099	0.109	−0.346	−0.684; 0.114	0.124
ACL - QoL - lifestyle	**−0.475**	−0.758; −0.041	**0.029**	−0.164	−0.566; 0.301	0.478
ACL - QoL - sport	**−0.576**	−0.812; −0.179	**0.006**	−0.174	−0.573; 0.271	0.450
ACL - QoL total score	**−0.467**	−0.760; −0.017	**0.038**	−0.334	−0.684; 0.141	0.150
Global Knee Function	0.166	−0.312; 0.567	0.485	0.238	−0.242; 0.624	0.312
ACL - RSI	−0.395	−0.720; 0.072	0.085	−0.245	−0.629; 0.235	0.299

Bold characters indicate a statistically significant correlation (p≤0.05).

POE = Postural Orientation Error, PROM = Patient-Reported Outcome Measures, K-SES = Knee Self-Efficacy Scale KOOS = Knee injury Osteoarthritis Outcome Score, ADL ​= ​Activity of Daily Living, QoL ​= ​Quality of Life, ACL-RSI ​= ​Anterior Cruciate Ligament–Return to Sport after Injury bold characters indicate statistically significant correlations.

## 4 Discussion

The result from this study indicates moderate to large associations between worse POE scores at baseline and worse scores on the K-SES and ACL- QoL questionnaires 2 years later, whereas no associations with KOOS, ACL-RSI, and global knee function were observed. Postural orientation during the rehabilitation phase may, thus, be important for future knee self-efficacy and knee-related QoL after ACLR, although further studies with larger sample size are needed to confirm these findings.

Extending the result from previous studies suggesting that adequate movement quality after ACL injury may be important for hop performance [13,14] and future activity level [12], we found moderate to large association between worse POE scores (subscale sport) during the rehabilitation phase and lower future knee self-efficacy and knee-related QoL. In a previous study on the same cohort, no cross-sectional associations were found between POEs and PROMs, including K-SES and ACL-QoL, seven moths post ACLR [13]. This indicates that postural orientation during the rehabilitation phase may be more important for future than current patient-reported outcomes. It may be speculated that factors such as strength and functional performance are more relevant for the individuals’ perceived function in the rehabilitation phase [26] but that postural orientation gain greater importance long-term. Strength and function improve during the course of the rehabilitation, whereas postural orientation may still be impaired [27]. Postural orientation, specifically during more demanding tasks such as hop tests, may thus be an important aspect to consider during the rehabilitation to improve future patient-reported outcomes. Future studies are, however, needed to confirm this assumption.

We found no associations between POEs during the rehabilitation phase and any of the KOOS subscales or scores on the ACL-RSI questionnaire two years post ACLR in the current study. This is in contrast to previous studies reporting those with poor movement quality at the time of RTS/3 years after ACL-injury to have worse KOOS scores two years later [12,15]. The previous studies did, however, use a test battery that includes several constructs, such as postural orientation, balance, body weight distribution and stride length [12], or assessed trunk and knee flexion asymmetries with 3D analysis [15], compared different time points (3 years [12] or time of RTS [15]) and included individuals both with and without ACLR [12]. These differences in population and methodology may explain the dissimilar results between those studies and the current study. It is possible that for example balance and/or asymmetries between the injured and non-injured leg are more important for future perceived function, as assessed with the KOOS, than frontal plane postural orientation on the injured leg only. On the other hand, in the current study, many of the correlations between POEs at baseline and the KOOS subscales as well as ACL-RSI at two years were between 0.3 and 0.4 but did not reach statistical significance. This indicates a potential relationship also between postural orientation and future knee-related function, as assessed with the KOOS subscales, as well as psychological readiness to RTS, that may have been masked by the small sample size (n ​= ​21) in the current study. Also, a previous analysis of this cohort revealed that women exhibited worse POEs compared with men [13]. Due to the low response rate in the current study, it was not possible to perform separate analyses for men and women. Hence, we do not know if the association between POEs and future PROMs is similar for both sexes. Future studies with a larger sample are therefore warranted to fully understand any possible associations between postural orientation and future PROMs in men and women after ACL injury.

POE sport was the only subscale that was associated with future PROMs in the current study, whereas there were no associations between the POE subscale ADL and any of the PROMs. The subscale sport is a sum of the POEs exhibited during the two hop tests (single-leg hop for distance, side-hop) and it is possible that perceived knee self-efficacy and knee-related QoL are more likely to be associated with the performance during such tasks in a population active in sports, such as the population in the current study (median pre-injury Tegner ​= ​8), compared to less demanding executed tasks reflecting activities of daily living.

This is the first longitudinal study evaluating possible associations between postural orientation during the rehabilitation phase after ACLR and a comprehensive set of future PROMs. The study is, nevertheless, associated with several limitations. First, this study was a secondary, explorative analysis of a previous cohort-study investigating measurement properties of the included test battery for evaluating POEs [19] and an a-priory sample size calculation was, thus, not performed. Second, only 40% of the participants answered the questionnaires at the 2-year follow-up, which constitutes a major limitation of this study. The low response rate could possibly be explained by the time taken to fill in the comprehensive set of PROMS that was administered to the participants [28]. There were, however, no differences in characteristics or baseline POEs between those who responded and those lost to follow-up limiting the effect of the response rate on the results. The low number of included participants may explain that several of the moderate correlations between POEs and PROMs did not reach statistical significance and we suggest that the result of this study may be used for hypothesis generating purposes for future studies with larger sample size. It should also be recognized that most of the correlations were only moderate, indicating that they only explain a part of the variation in PROMs. Third, we did not adjust for muscle function, such as hop performance or muscle strength at baseline or at the two-year follow-up. People with ACL injury seem still to have impairments in hop performance and muscle strength two years after ACLR [29]. Furthermore, worse performance on hop tests have previously been related to worse scores on the KOOS subscales [12] and lower knee muscle strength has been reported to contribute to perceived knee self-efficacy [30]. It is, therefore, possible that the result would have been different if we were able to take other measures of muscle function into account. Along the same line, we did not assess POEs at the follow-up, hence we cannot rule out that the result of this study may be attributed to POEs (or other functional factors) exhibited at the time of filling in the questionnaires rather than baseline POEs.

## 5 Conclusion

Postural orientation during hop tests in patients undergoing rehabilitation for ACLR may be important for future knee self-efficacy and knee-related QoL. Future studies with a larger sample are, however, warranted to confirm this result.

## Availability of data and materials

The data used in this study contains sensitive information about the study participants and they did not provide consent for public data sharing. The current approval by the Swedish Ethical Review Board does not include data sharing. A minimal data set could be shared by request from a qualified academic investigator for the sole purpose of replicating the present study, provided the data transfer is in agreement with EU legislation on the general data protection regulation and approval by the Swedish Ethical Review Authority.

## Authors’ contribution

Each of the authors was fully involved in the study and preparation of the manuscript, including final review of the submitted work.

## Ethical compliance

The study was approved by the Regional Ethical Review Board in Lund, Sweden (2018/14) and all participants gave their written informed consent.

## Funding

No funding was received specifically for this study.

## Declaration of competing interest

The authors declare that they have no known competing financial interests or personal relationships that could have appeared to influence the work reported in this paper.
