# Supplementary material for: Associations between postural orientation errors in patients undergoing rehabilitation for ACL reconstruction and future patient-reported outcomes: An explorative study
Source: JSAMS Plus. 2023 Sep 24;2:100039. doi: 10.1016/j.jsampl.2023.100039 (PMC13008436; doi:10.1016/j.jsampl.2023.100039)
Supplement: Multimedia component 1 [file mmc1.docx]

# APPENDIX A

| **Table 1.** The test battery of tasks and POEs assessed within each task, the calculations for the percentage scale and median (quartiles) for each task and POE sub scale scores. | | | | | | | | |
| --- | --- | --- | --- | --- | --- | --- | --- | --- |
| **Functional tasks** | **Ankle POE** | **Knee POEs** | | **Thigh POE** | **Hip POEs** | | **Trunk POEs** | **Within-task POE score** |
|  | **Foot pronation** | **Knee Medial-to-foot position** | **Femur medial to shank** | **Femoral valgus** | **Deviation of pelvis in any plane** | | **Deviation of trunk in any plane** |  |
| Single-leg mini squat | X | X | X | X | X | | X | $\frac{sum score}{18}x 100$ |
| Stair descending |  |  | X | X |  | |  | $\frac{sum score}{6}x 100$ |
| Forward lunge |  | X | X | X | X | |  | $\frac{sum score}{12}x 100$ |
| Single-leg hop for distance |  | X | X | X | X | |  | $\frac{sum score}{12}x 100$ |
| Side-hop  lateral landing |  | X | X | X |  | |  | $\frac{sum score}{24}x 100$ |
| Side-hop medial landing |  | X | X | X | X | | X |  |
| Subscale  ADL | (Sum score of single-leg mini squat, stair descending and forward lunge) | | | | | $\frac{sum score}{36}x100$ | | |
| Subscale Sport | (Sum score of single-leg hop for distance and side hop) | | | | | $\frac{sum score}{36}x100$ | | |

This table is adapted from a previously published study ^1^. POE = Postural Orientation Errors, ADL = Activity of Daily Living

1. Nae J, Creaby MW, Nilsson G, Crossley KM, Ageberg E. Measurement Properties of a Test Battery to Assess Postural Orientation During Functional Tasks in Patients Undergoing Anterior Cruciate Ligament Injury Rehabilitation. *J Orthop Sports Phys Ther.* 2017;47(11):863-873.
