# Supplementary material for: Associations between postural orientation errors in patients undergoing rehabilitation for ACL reconstruction and future patient-reported outcomes: An explorative study
Source: JSAMS Plus. 2023 Sep 24;2:100039. doi: 10.1016/j.jsampl.2023.100039 (PMC13008436; doi:10.1016/j.jsampl.2023.100039)
Supplement: Multimedia component 2 [file mmc2.docx]

**Appendix B**

Scatterplots for correlations between POEs at baseline and patient-reported outcome measures at 2 year follow-up (women n=7, men n=14).


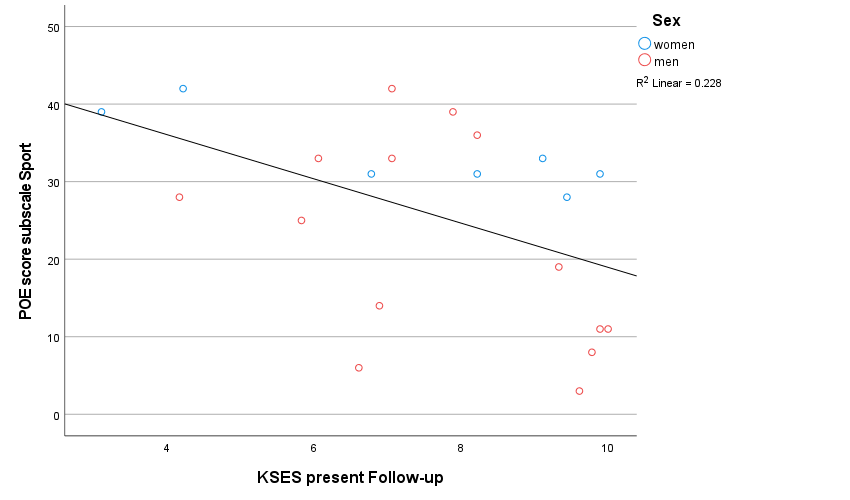


**Figure 1.** Correlation between postural orientation error (POE) scores subscale sport at baseline and the Knee Self-Efficacy Scale (KSES) at 2 year follow-up


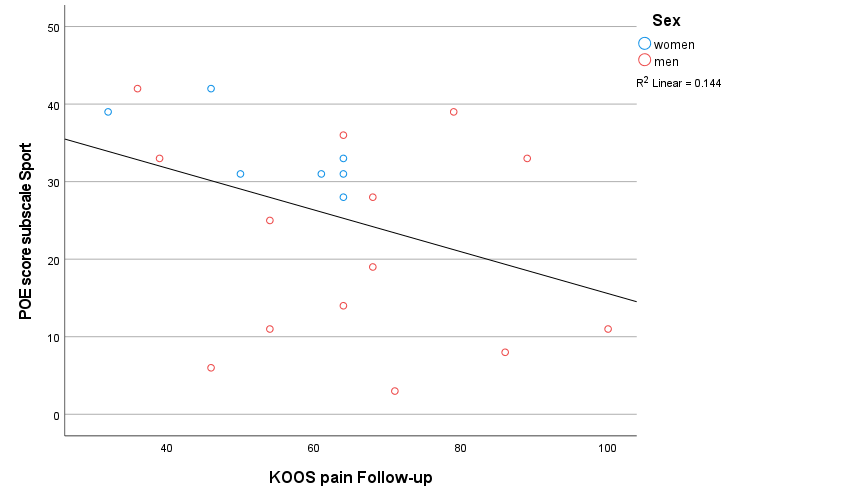


**Figure 2.** Correlation between postural orientation error (POE) scores subscale sport at baseline and the Knee injury and Osteoarthritis Outcome Score (KOOS) subscale pain at 2 year follow-up


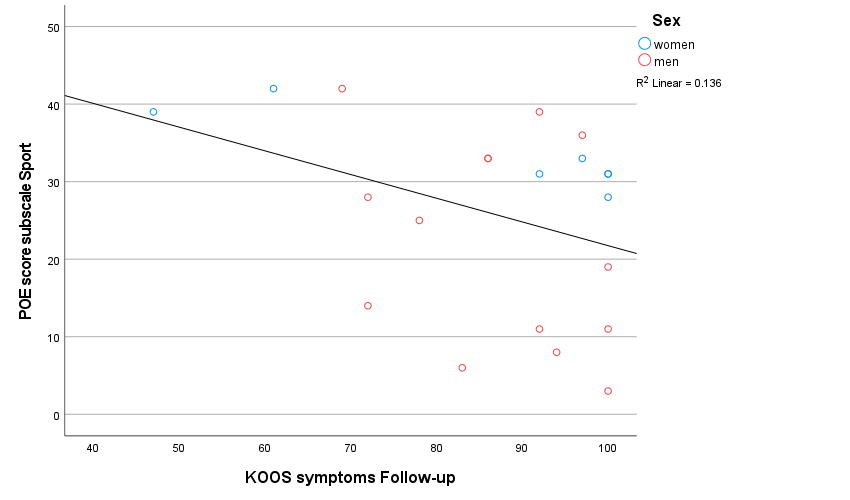


**Figure 3.** Correlation between postural orientation error (POE) scores subscale sport at baseline and the Knee injury and Osteoarthritis Outcome Score (KOOS) subscale symptoms at 2 year follow-up


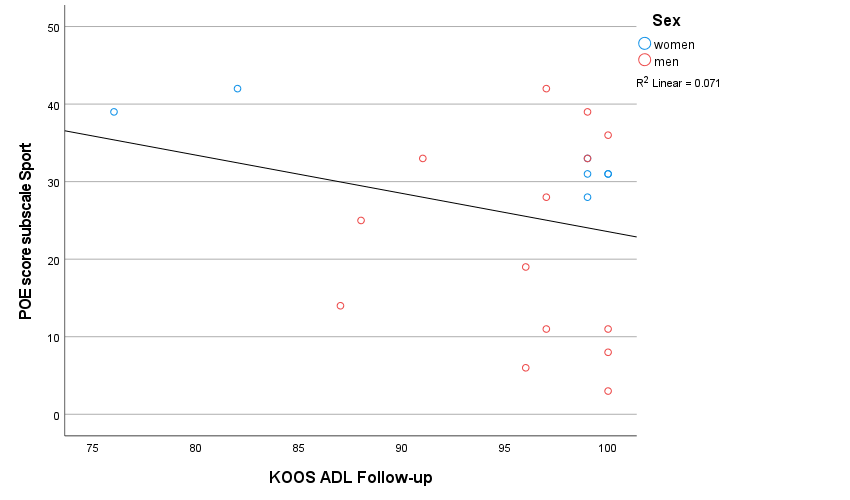


**Figure 4.** Correlation between postural orientation error (POE) scores subscale sport at baseline and the Knee injury and Osteoarthritis Outcome Score (KOOS) subscale activity of daily living (ADL) at 2 year follow-up


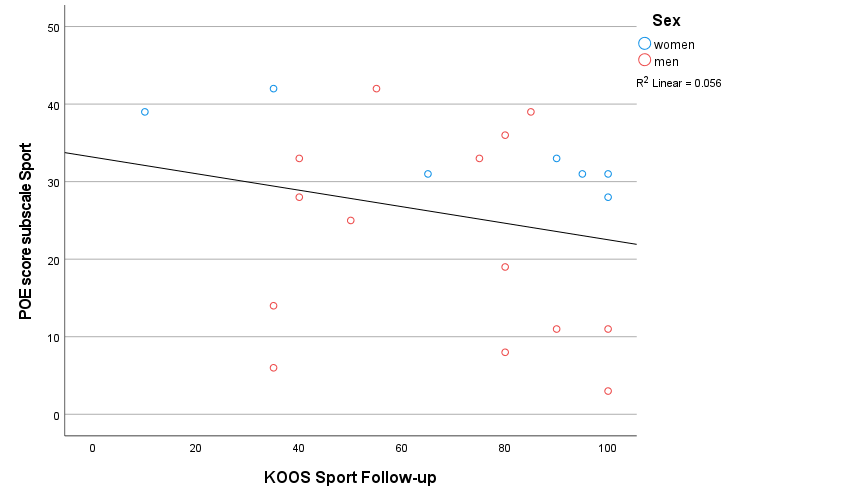


**Figure 5.** Correlation between postural orientation error (POE) scores subscale sport at baseline and the Knee injury and Osteoarthritis Outcome Score (KOOS) subscale sport at 2 year follow-up


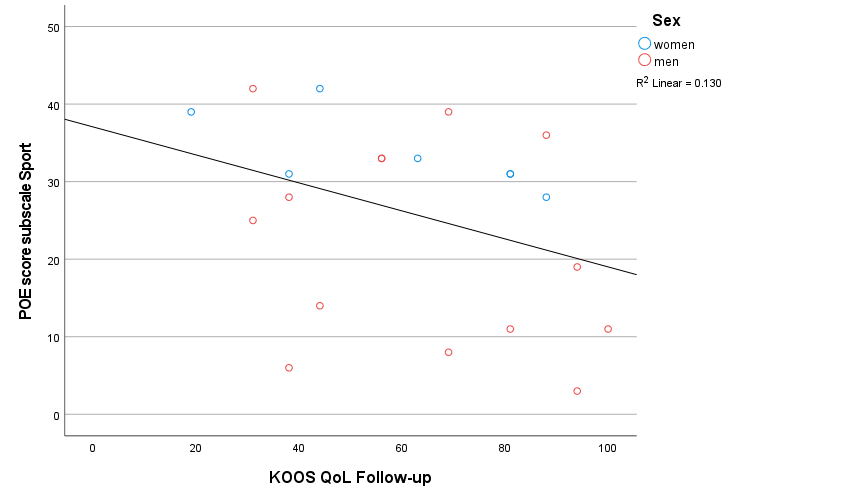


**Figure 6.** Correlation between postural orientation error (POE) scores subscale sport at baseline and the Knee injury and Osteoarthritis Outcome Score (KOOS) subscale quality of life (QoL) at 2 year follow-up


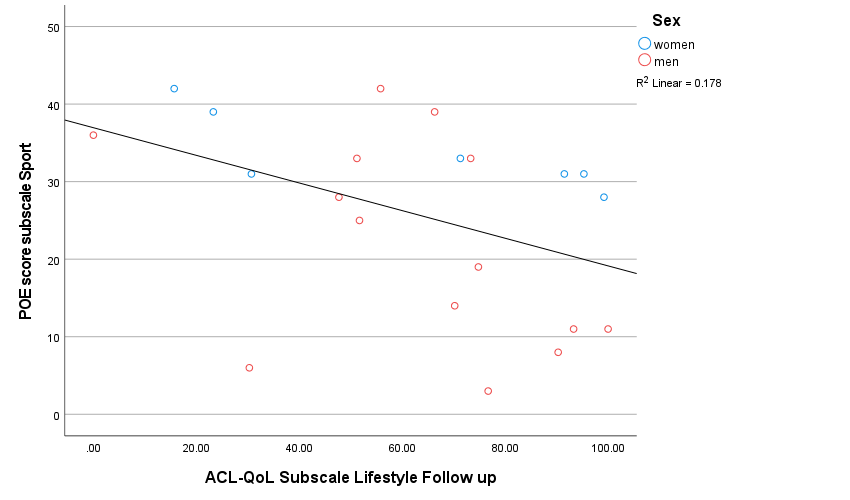


**Figure 7.** Correlation between postural orientation error (POE) scores subscale sport at baseline and the Anterior Cruciate Ligament-Quality of Life (ACL-QoL) subscale lifestyle at 2 year follow-up


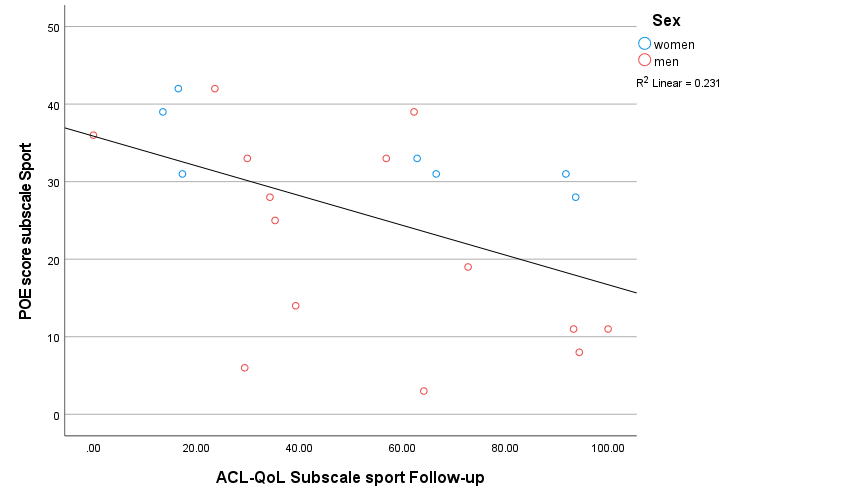


**Figure 8.** Correlation between postural orientation error (POE) scores subscale sport at baseline and the Anterior Cruciate Ligament-Quality of Life (ACL-QoL) subscale sport at 2 year follow-up


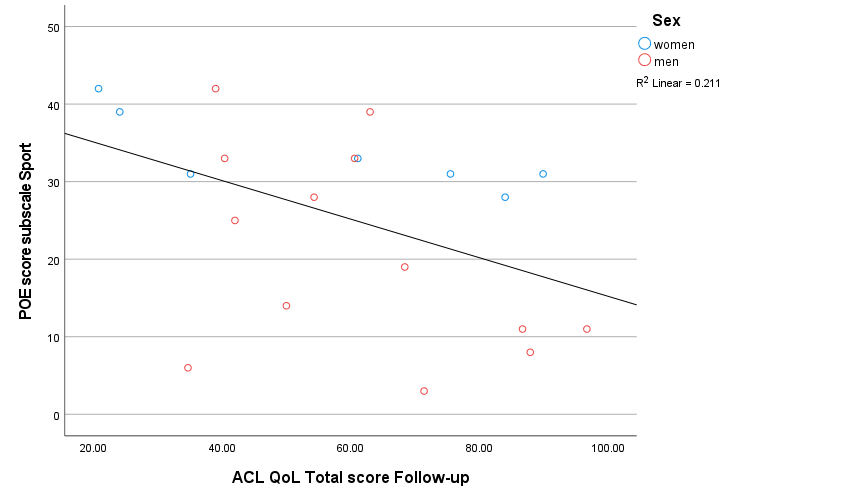


**Figure 9.** Correlation between postural orientation error (POE) scores subscale sport at baseline and the Anterior Cruciate Ligament-Quality of Life (ACL-QoL) total score at 2 year follow-up


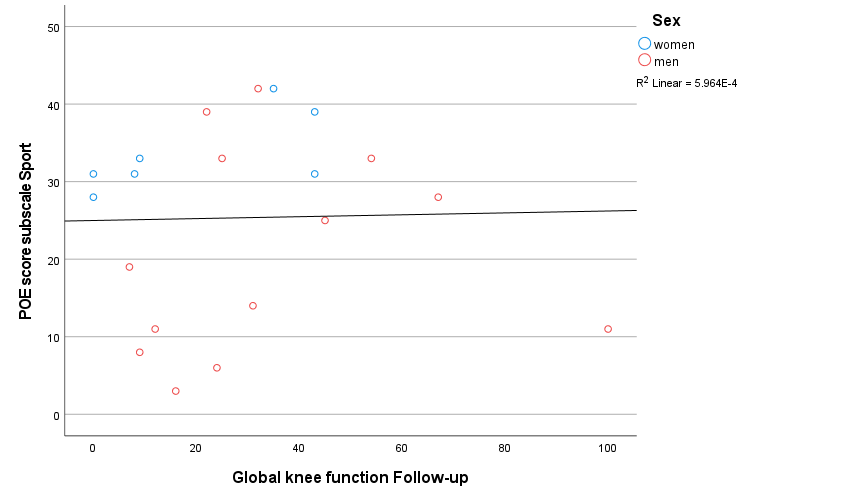


**Figure 10.** Correlation between postural orientation error (POE) scores subscale sport at baseline and global knee function at 2 year follow-up


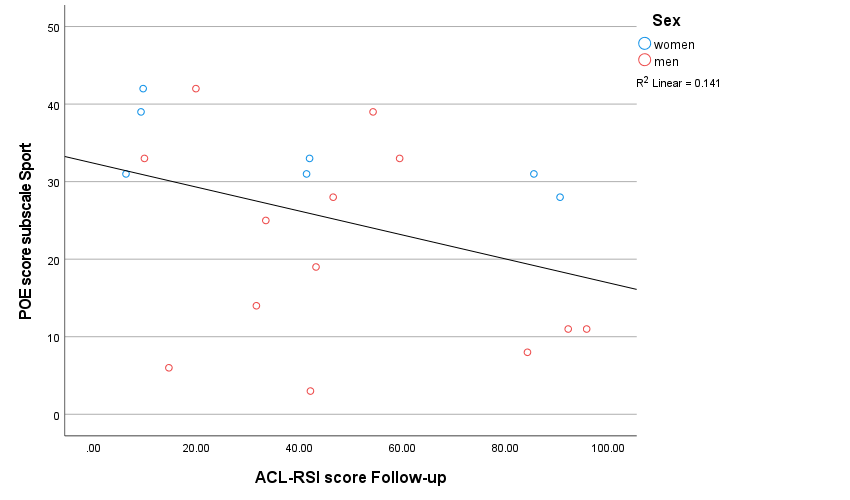


**Figure 11.** Correlation between postural orientation error (POE) scores subscale sport at baseline and the Anterior Cruciate Ligament–Return to Sport after Injury (ACL-RSI) scale at 2 year follow-up


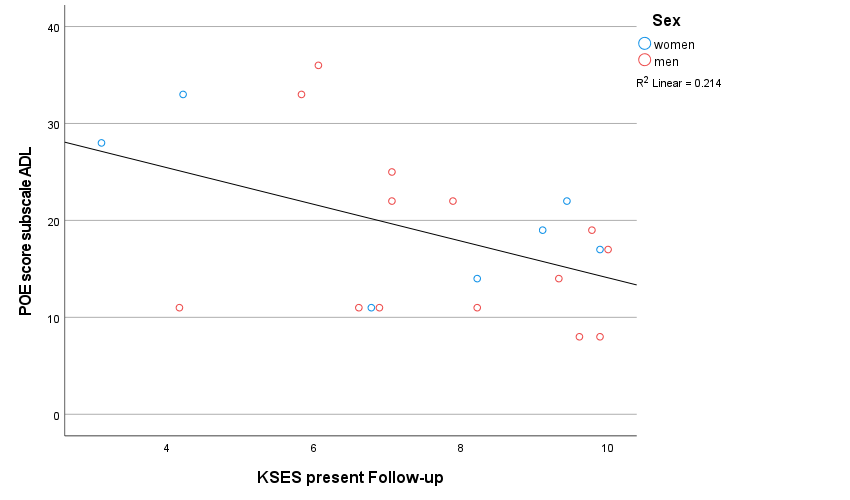


**Figure 12.** Correlation between postural orientation error (POE) scores subscale activity of daily living (ADL) at baseline and the Knee Self-Efficacy Scale (KSES) at 2 year follow-up


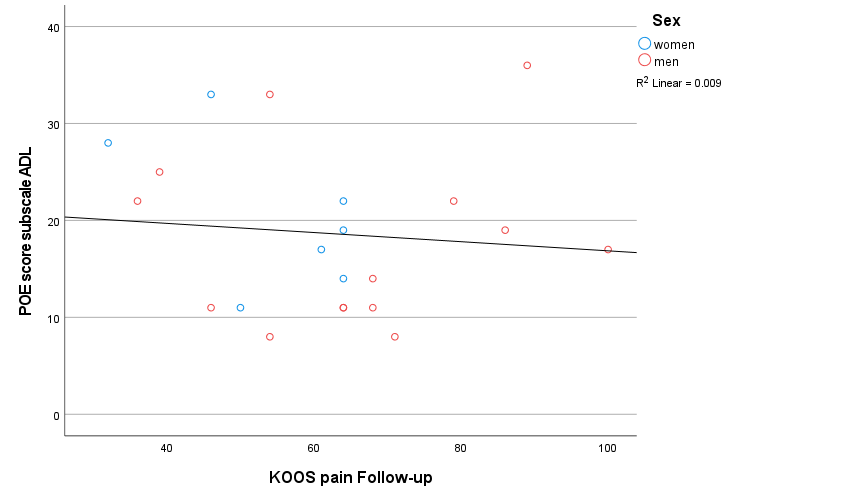


**Figure 13.** Correlation between postural orientation error (POE) scores subscale activity of daily living (ADL) at baseline and the Knee injury and Osteoarthritis Outcome Score (KOOS) subscale pain at 2 year follow-up


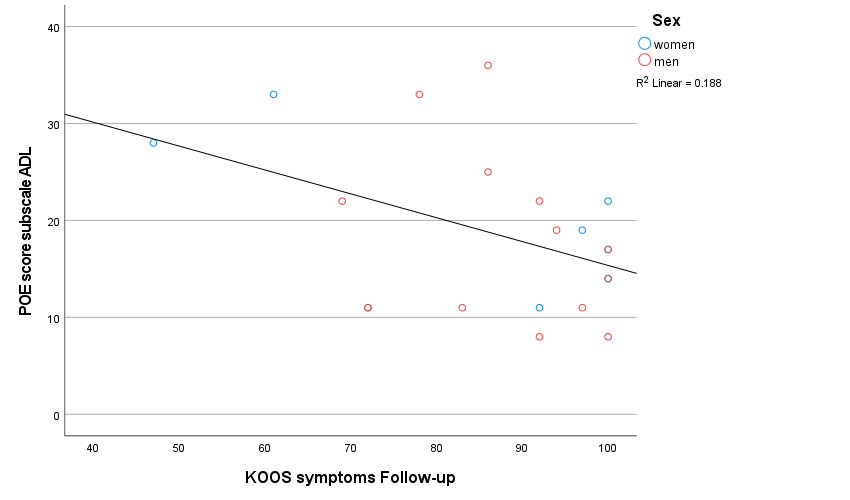


**Figure 14.** Correlation between postural orientation error (POE) scores subscale activity of daily living (ADL) at baseline and the Knee injury and Osteoarthritis Outcome Score (KOOS) subscale symptom at 2 year follow-up


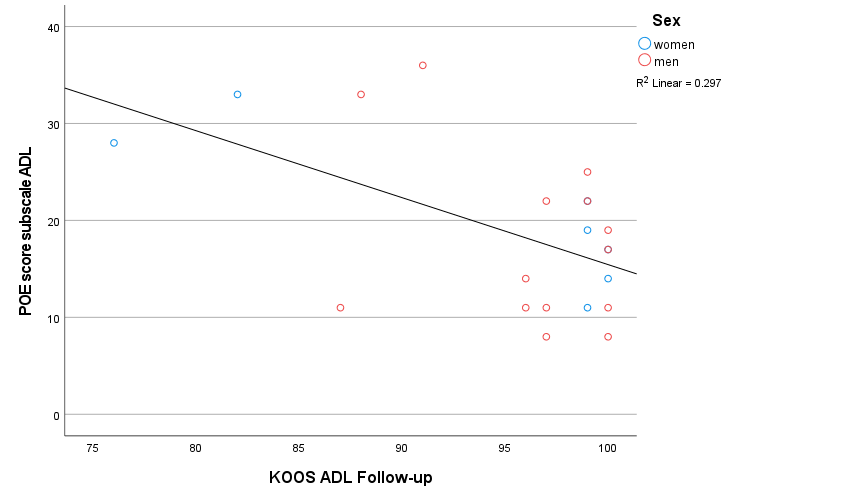


**Figure 15.** Correlation between postural orientation error (POE) scores subscale activity of daily living (ADL) at baseline and the Knee injury and Osteoarthritis Outcome Score (KOOS) subscale activity of daily living (ADL) at 2 year follow-up


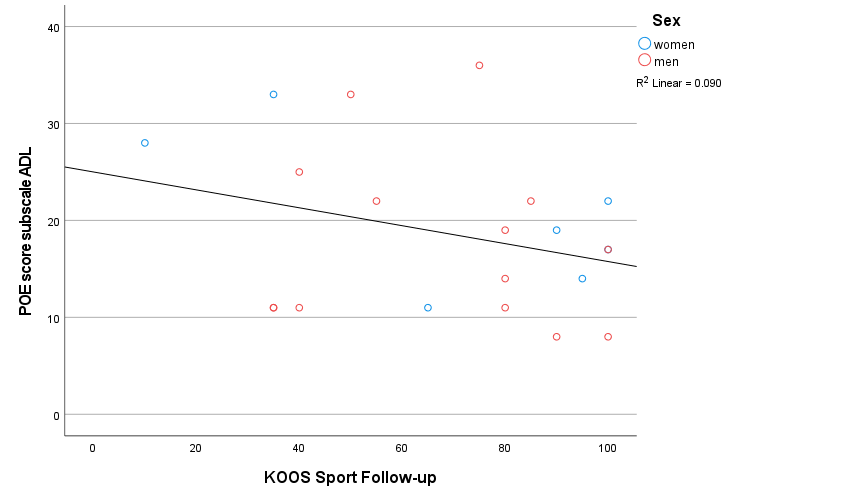


**Figure 16.** Correlation between postural orientation error (POE) scores subscale activity of daily living (ADL) at baseline and the Knee injury and Osteoarthritis Outcome Score (KOOS) subscale sport at 2 year follow-up


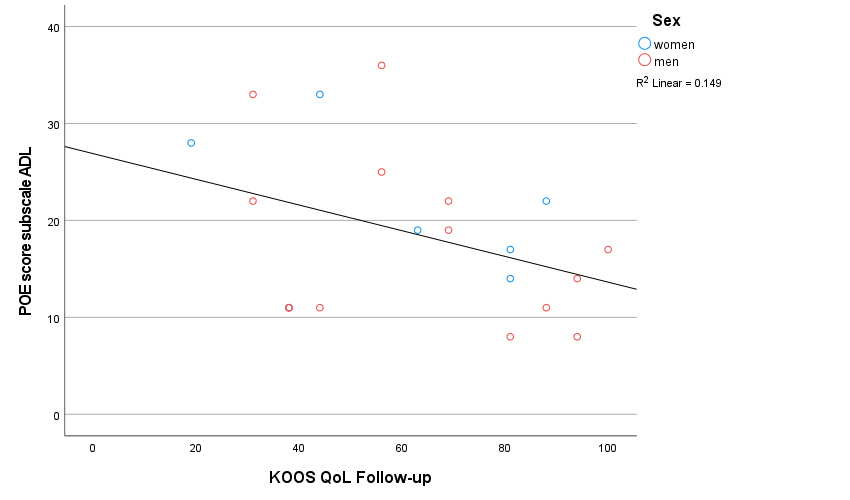


**Figure 17.** Correlation between postural orientation error (POE) scores subscale activity of daily living (ADL) at baseline and the Knee injury and Osteoarthritis Outcome Score (KOOS) subscale quality of life (QoL) at 2 year follow-up


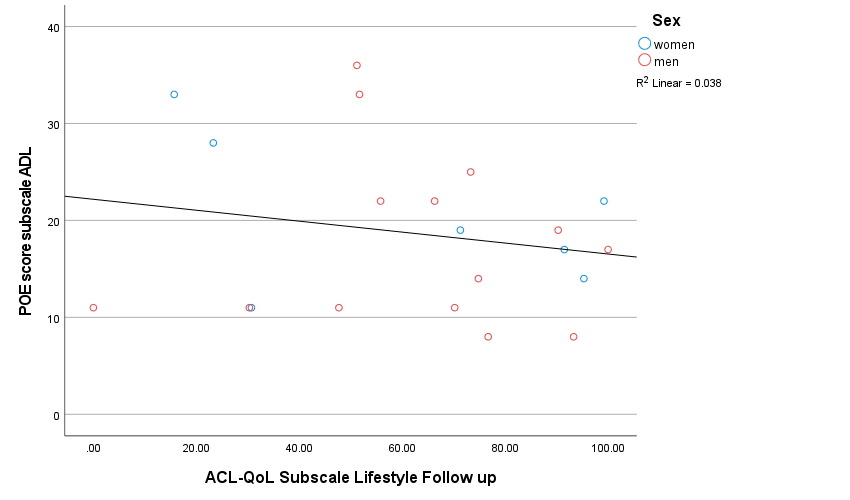


**Figure 18.** Correlation between postural orientation error (POE) scores subscale activity of daily living (ADL) at baseline and the Anterior Cruciate Ligament-Quality of Life (ACL-QoL) subscale lifestyle at 2 year follow-up


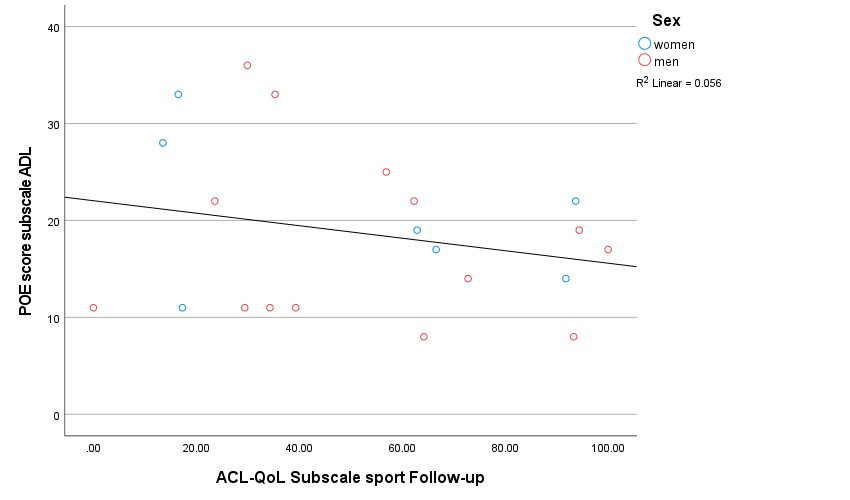


**Figure 19.** Correlation between postural orientation error (POE) scores subscale activity of daily living (ADL) at baseline and the Anterior Cruciate Ligament-Quality of Life (ACL-QoL) subscale sport at 2 year follow-up


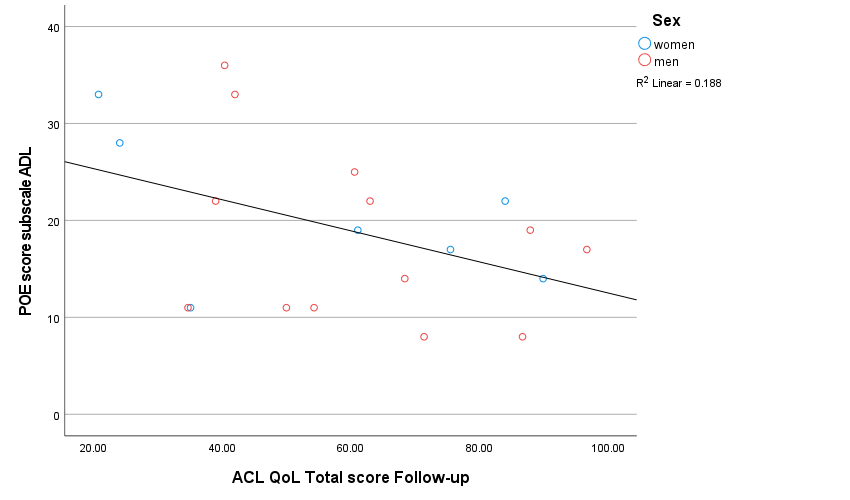


**Figure 20.** Correlation between postural orientation error (POE) scores subscale activity of daily living (ADL) at baseline and the Anterior Cruciate Ligament-Quality of Life (ACL-QoL) total score at 2 year follow-up


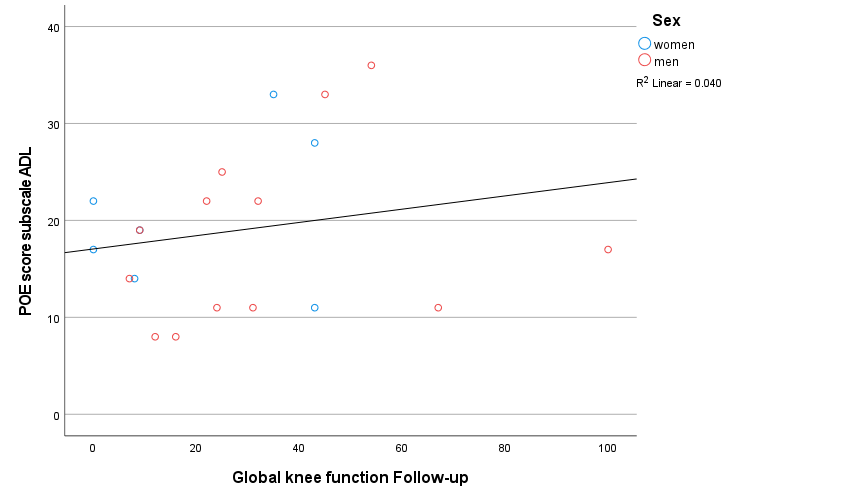


**Figure 21.** Correlation between postural orientation error (POE) scores subscale activity of daily living (ADL) at baseline and global knee function at 2 year follow-up


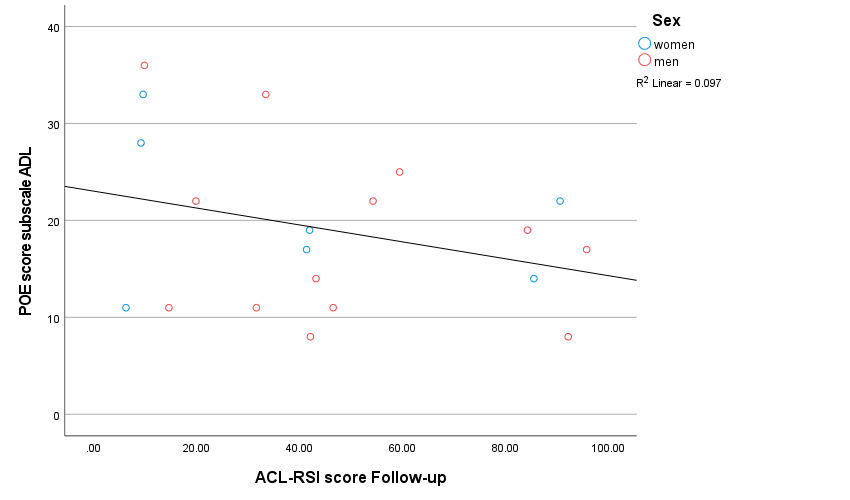


**Figure 22.** Correlation between postural orientation error (POE) scores subscale activity of daily living (ADL) at baseline and Anterior Cruciate Ligament–Return to Sport after Injury (ACL-RSI) scale at 2 year follow-up
